# An Unusual Location of Subungual Warty Dyskeratoma: A Case Report and Review of the Literature

**DOI:** 10.1155/2017/3613109

**Published:** 2017-02-23

**Authors:** Elena Vargas-Laguna, Adrián Imbernón-Moya, Antonio Aguilar-Martínez, Fernando Burgos

**Affiliations:** ^1^Department of Dermatology, Hospital Universitario Severo Ochoa, Avenida de Orellana, Leganés, 28911 Madrid, Spain; ^2^Department of Pathology, Hospital Universitario Severo Ochoa, Avenida de Orellana, Leganés, 28911 Madrid, Spain

## Abstract

Warty dyskeratoma is an uncommon entity characterized by a solitary keratotic papule or nodule usually located in the head and neck of young adults. The histopathology shows a pattern of acantholytic dyskeratosis. We report a 32-year-old man who presented pain, serous exudation, a distal onycholysis with subungual hyperkeratosis, and roundish erythronychia in the nail plate of his left first toe 2 years ago. A histopathologic diagnosis of subungual warty dyskeratoma was made. When dealing with focal acantholytic dyskeratosis several differential diagnoses should be considered including Darier's disease, transient focal acantholytic dyskeratosis or Grover disease, and Hailey-Hailey disease. We present an unusual location of warty dyskeratoma in the nail bed using a clinicohistopathological correlation for the diagnosis.

## 1. Introduction

Warty dyskeratoma (WD) is an unusual benign tumour with a follicular origin. It usually appears as a solitary reddish-brown papule, but cases of multiple lesions have been observed. The typical location is in the head and neck of young adults; nevertheless isolated cases of warty dyskeratoma have been reported on the oral mucosa and on the vulva [1–3].

## 2. Case Presentation

**Figure 1:**
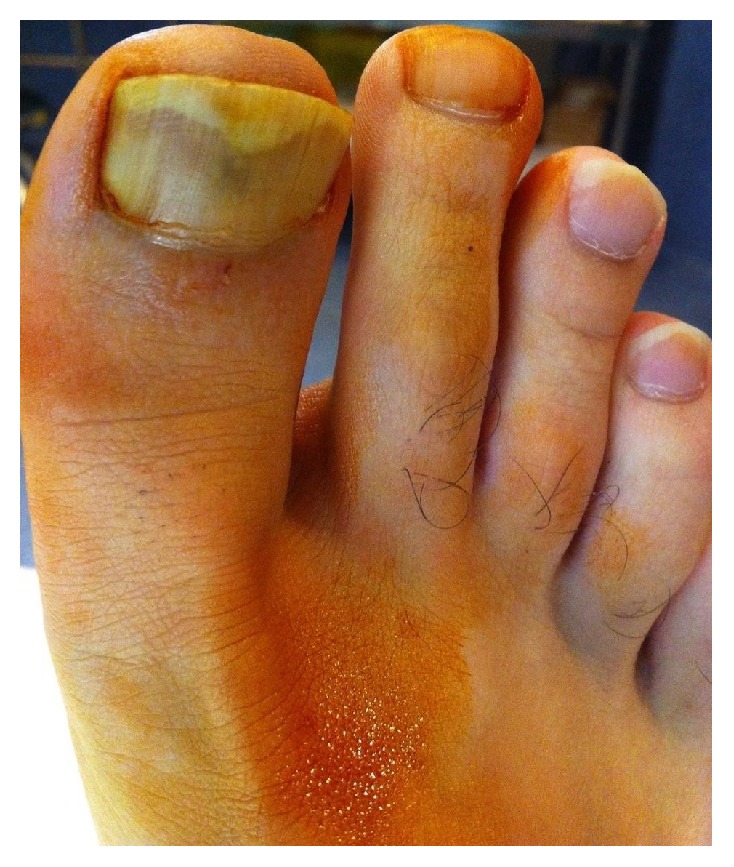
Subungual hyperkeratosis and a round reddish area in the middle of the nail plate.

We present a Caucasian 32-year-old man, with no personal or family history of disease, who was referred to the Dermatology Department due to two-year history of pain and serous exudation on the nail of his left first toe. An avulsion of his nail was performed one year ago but there has been no visible improvement. The patient denied prior trauma or any regular medication. Dermatological examination showed a distal onycholysis with subungual hyperkeratosis and a round reddish area in the middle of the nail plate (Figure 1). The remaining nails did not show any abnormalities. An X-ray and a magnetic resonance imaging did not show any sign of lesions.

**Figure 2:**
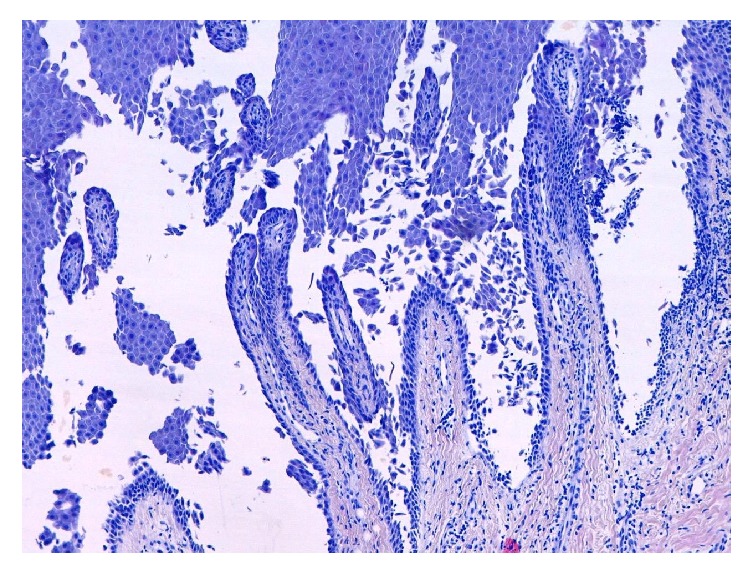
Suprabasal acantholytic and dyskeratotic keratinocytes (H-E ×10).

An avulsion of the nail plate revealed an eight mm reddish papule in the middle of the nail bed. A sample specimen of this papule was taken. The histologic features showed acantholytic phenomenon in the epidermis, dyskeratotic keratinocytes, hyperkeratosis, and bone of the distal phalanx (Figure 2).

## 3. Discussion

Warty dyskeratoma is an uncommon benign tumour first reported by Helwig in 1954 as “isolated Darier's disease” [1]. It usually appears as an asymptomatic solitary reddish-brown papule or nodule, with a keratotic umbilicate centre, located in the head and neck of young adults. Multiple lesions and several cases located on oral mucosa have been reported [2, 3]. The histopathology shows a characteristic pattern of acantholytic dyskeratosis that is not pathognomonic. The treatment of choice is surgical excision. Curettement with electrodesiccation has been reported as well as topical tazarotenic acid [4].

When dealing with focal acantholytic dyskeratosis (FAD) several differential diagnoses should be considered. Typically it is a distinctive histological feature of Darier's disease, transient focal acantholytic dyskeratosis or Grover disease, and Hailey-Hailey disease [3]. Nevertheless FAD has been observed as an incidental finding in several skin lesions such as compound and junctional nevi, scars, seborrheic keratosis, basal cell carcinoma, and squamous cell carcinoma [5].

Another entity that shows acantholytic dyskeratosis is the acantholytic dermatosis of the vulvocrural area. It typically presents as multiple papules on the perineum, inguinal folds, vulva, and perianal sites [6]. The intertriginous acantholytic dermatosis is a different clinical entity from Haley-Haley disease and intertriginous Darier's disease, although similar histopathological changes can be found.

Ackerman suggested that the term WD should be reserved for lesions that appear as a single nodule but not as a papule, for which he suggested the term “papular form of FAD” [7]. However other authors continue using the term WD for both clinical patterns.

The aetiology is unknown but a few etiopathogenic factors were postulated including trauma, ultraviolet radiations, tobacco, and viral infections.

Baran reported a subungual dyskeratoma in the fingernail, with the clinical appearance of longitudinal erythronychia. Histological features showed deep epithelial digitations in the nail bed that lined a crater-like area with intense papillomatosis at the base of the crater and acantholytic suprabasal clefts inside the tumour. Furthermore numerous multinucleated giant cells were present in the nail bed [8].

Isonokami and Higashi reported another case of subungual warty dyskeratoma in a young woman's fingernail, accompanied with pain. A 2 mm longitudinal reddish ridge was seen in the nail plate. The histopathological changes presented in the nail matrix and in the nail bed showed a crater-like area with irregulars cleft, acantholysis, and dyskeratotic cells [9].

Erythronychia, especially red longitudinal streaks in the nail plate, is usually associated with glomus tumour, Bowen's disease, warty dyskeratoma, onychopapilloma, and melanoma in rare cases [10].

In conclusion, we report an unusual location of WD in the nail bed using a clinicohistopathological correlation for the diagnosis. As a peculiarity of this case, the location of the lesion in the nail bed produces a roundish erythronychia while lesions located in the nail matrix could develop longitudinal erythronychia. Although WD is described as a solitary tumour, we consider that the cause of this histological finding has not been determined.

## Acronyms

WD: Warty dyskeratoma
FAD: Focal acantholytic dyskeratosis.
